# Central Interleukin-1*β* Suppresses the Nocturnal Secretion of Melatonin

**DOI:** 10.1155/2016/2589483

**Published:** 2016-04-26

**Authors:** A. P. Herman, J. Bochenek, K. Król, A. Krawczyńska, H. Antushevich, B. Pawlina, A. Herman, K. Romanowicz, D. Tomaszewska-Zaremba

**Affiliations:** ^1^The Kielanowski Institute of Animal Physiology and Nutrition, Polish Academy of Sciences, Instytucka 3 Street, 05-110 Jabłonna, Poland; ^2^The Academy of Cosmetics and Health Care, 13 Podwale Street, 00-252 Warsaw, Poland

## Abstract

In vertebrates, numerous processes occur in a rhythmic manner. The hormonal signal reliably reflecting the environmental light conditions is melatonin. Nocturnal melatonin secretion patterns could be disturbed in pathophysiological states, including inflammation, Alzheimer's disease, and depression. All of these states share common elements in their aetiology, including the overexpression of interleukin- (IL-) 1*β* in the central nervous system. Therefore, the present study was designed to determine the effect of the central injection of exogenous IL-1*β* on melatonin release and on the expression of the enzymes of the melatonin biosynthetic pathway in the pineal gland of ewe. It was found that intracerebroventricular injections of IL-1*β* (50 *µ*g/animal) suppressed (*P* < 0.05) nocturnal melatonin secretion in sheep regardless of the photoperiod. This may have resulted from decreased (*P* < 0.05) synthesis of the melatonin intermediate serotonin, which may have resulted, at least partially, from a reduced expression of tryptophan hydroxylase. IL-1*β* also inhibited (*P* < 0.05) the expression of the melatonin rhythm enzyme arylalkylamine-N-acetyltransferase and hydroxyindole-O-methyltransferase. However, the ability of IL-1*β* to affect the expression of these enzymes was dependent upon the photoperiod. Our study may shed new light on the role of central IL-1*β* in the aetiology of disruptions in melatonin secretion.

## 1. Introduction

In vertebrates, numerous processes occur in a rhythmic manner. During the course of evolution, animals have evolved biological rhythms that are associated with changes in the lighting and temperature of their environment. One of the most important biological rhythms is the circadian rhythm generated by endogenous oscillators, termed the “circadian clock,” which has a period very close to 24 hours and is synchronised with environmental light conditions. The hormonal signal used to reliably reflect environmental light conditions is melatonin (N-acetyl-5-methoxytryptamine), which is synthesised in the pineal gland [1]. This gland is localised in a third ventricle (IIIv) evagination called the pineal recess [2]. It stays in contact with the cerebrospinal fluid (CSF) from the IIIv, which results in the local concentration of melatonin in the IIIv being approximately 20 times higher in the CSF than in blood [3]. In all mammalian species studied, melatonin secretion is regulated by norepinephrine (NE), which is released from sympathetic nerve fibres exclusively at night [1]. The biosynthesis of melatonin is a well-characterised multistep sequence of reactions that starts with the hydroxylation of tryptophan to 5-hydroxytryptophan (5-HTP) by the enzyme tryptophan hydroxylase (TPH). Next, aromatic amino acid decarboxylase (DDC) converts 5-HTP to 5-hydroxytryptamine (5-HT; serotonin). Then, 5-HT is transformed to N-acetylserotonin by arylalkylamine-N-acetyltransferase (AANAT). Finally, N-acetylserotonin is converted to melatonin by the enzyme hydroxyindole-O-methyltransferase (HIOMT) [4]. The circulating level of melatonin reflects pineal secretory activity because it is not stored within the pinealocytes but freely diffuses out of the cells into blood capillaries immediately after its formation [1]. A conservative feature of vertebrates is that the plasma level of melatonin increases at night, whereas diurnal levels of this hormone are relatively low [5].

One of the most significant pleiotropic effects of melatonin is the regulation of the immune system. Both diurnal and seasonal changes in immune function are thought to directly reflect changes in pineal melatonin secretion. Melatonin plays a role as an immunomodulator, regulating the development, differentiation, and function of lymphoid tissues [6]. The pineal gland likely participates in the innate immune response because it expresses mRNA encoding transcripts for all ten members of the toll-like receptor (TLR) family. Therefore, the function of this gland may be affected by a number of the pathogen-associated molecular patterns recognised by these receptors [7]. The interactions occurring between the immune system and the pineal gland seem to be bidirectional. However, the feedback effect of the inflammatory response on the pineal gland's neuroendocrine functions is poorly understood. Molecules carrying feedback information from the immune system to the pineal gland may include inflammatory mediators, such as cytokines, prostaglandins (PGs), and histamine, that penetrate this region of brain during inflammatory response [8]. A few studies have shown that the secretory activity of pinealocytes can be modified by antigenic stimulation [9], histamines [10], cytokines [11, 12], and prostaglandins [13, 14]. Also our previous* ex vivo* study showed that the pleiotropic proinflammatory cytokine interleukin- (IL-) 1*β* suppressed melatonin secretion in ovine pineal glands [15]. Immune mediators have easy access to the pineal gland because it is a part of the brain that lacks the blood-brain barrier [16]. The secretory activity of the pineal gland may also be modulated by immune mediators present in the CSF because during peripheral inflammation proinflammatory cytokines, including IL-1*β*, IL-6, and tumor necrosis factor (TNF)*α* can cross the blood-brain and blood-CSF barriers and reach the brain parenchyma [17–19]. A study on rats showed that almost immediately after the peripheral injection of the bacterial endotoxin lipopolysaccharide (LPS), the level of proinflammatory cytokines in the CSF is significantly elevated [20]. Moreover, melatonin synthesis may be affected by the autocrine actions of inflammatory mediators, as it has been reported that, in addition to producing melatonin, the pineal gland can be induced to synthesise several cytokines, such as IL-1*β*, IL-6, and TNF [21–24]. Factors other than inflammation may influence pineal melatonin production via mechanisms that are dependent on proinflammatory cytokines. The decrease in melatonin secretion observed in association with Alzheimer's disease has been postulated as being responsible for the circadian disorganisation, decrease in sleep efficiency, and impaired cognitive function observed in patients with this disease [25]. Sleep difficulties and other symptoms associated with low melatonin levels also accompany depressive disorders [26, 27]. There is evidence implicating the levels of IL-1*β* in the brain in the aetiology and pathophysiology of both Alzheimer's disease and depression [28, 29].

The majority of the studies concerning the broadly understood bilateral interactions between the immune and pineal systems have been performed on rodents, mainly rats, which are nocturnal species. However, there are important differences in the intracellular mechanisms regulating melatonin secretion that distinguish rodents and other mammals, including ungulates and primates [1]. Therefore, the present studies were performed on ewes, which exhibit significantly greater similarities in their mechanisms regulating melatonin secretion to human than do rodent species.

This study was designed to determine the effects of IL-1*β* injected into the IIIv of the brain on melatonin release and the expression of the enzymes of the melatonin biosynthetic pathway in sheep during two distinct photoperiodic conditions: a short night (SN-long-day; 8 : 16) period and long night (LN-short-day; 16 : 8) period.

## 2. Materials and Methods

### 2.1. Animals and Experimental Design

These studies were performed on adult, two-year-old blackface ewes during two different photoperiods: in May/June during a short night (long-day, 8 : 16) period and in November/December during long night (short-day, 16 : 8) period. The experiments started one month before the summer and winter solstices, respectively. The animals were maintained indoors in individual pens and were exposed to the natural daylight present at 52°N latitude and 21°E longitude. The ewes were in good condition; that is, their body condition was estimated to be 3 based on a five-point scale [30], and the animals were acclimated to the experimental conditions for one month. The ewes were always within visual contact with other members of the flock to prevent isolation stress. The animals were fed a consistent diet of commercial concentrates with hay and water available* ad libitum*. One month before the experiment, all groups of ewes were cannulated with push-pull cannulas into the third ventricle according to the method described elsewhere [31]. All animals had venous catheters implanted into their jugular vein the day before the experiment. All procedures were performed with the consent of the Local Ethics Committee of Warsaw University of Life Sciences, SGGW Poland.

In each of the two experimental treatments, the animals (*n* = 12) were randomly divided into two groups: control (*n* = 6) and IL-1*β*-treated (*n* = 6) ewes. The experimental procedures were performed during the night, in the darkness, and in the presence of a red light. Two hours after sunset, the animals received an intracerebroventricular (icv.) injection of IL-1*β* (50 *μ*g/animal; Sigma-Aldrich, St. Louis, MO, USA) dissolved in 50 *μ*L of Ringer's solution through stainless steel cannulas. Control animals received only 50 *μ*L Ringer's solution. The blood samples were then collected at 15 min intervals, starting one hour after sunset and continuing for 5 hours after the treatment. Blood samples were collected into heparinised tubes and immediately centrifuged in an MPW 260RH centrifuge (MPW Med. Instruments, Warsaw, Poland) for 10 min at 1000 ×g at 4°C. Plasma was stored at −80°C until assayed. The animal's body temperature was measured at one-hour intervals. After two weeks of convalescence, the animals received the same treatments again. The ewes were euthanised 4 hours after the final central injection of IL-1*β* or Ringer's solution at 3 a.m. during the LD period and 10:00 p.m. during SD period. Their brains were immediately removed from their skulls, and the pineal gland was dissected into two parts, frozen in liquid nitrogen, and stored at −80°C until assay.

### 2.2. Assays

#### 2.2.1. Radioimmunoassay of Plasma Hormones

Melatonin was assayed in the blood plasma according to the method of Fraser et al. [32] and as modified in our laboratory using ovine antimelatonin serum (Dr. A. Foldes, CSRIO, Australia). Synthetic melatonin (Sigma-Aldrich, St. Louis, MO, USA) was used as a standard and [O-methyl-3H]-melatonin (Amersham PLC, Amersham, UK) as a tracer. The sensitivity of the assay was 17 ± 8 pg/mL and the intra- and interassay coefficients of variation were 11% and 13%, respectively.

The cortisol concentrations in the plasma were determined via radioimmunoassay according to Kokot and Stupnicki [33], using rabbit anti-cortisol antisera (R/75) and an HPLC-grade cortisol standard (Sigma-Aldrich, St. Louis, MO, USA). The assay sensitivity was 1 ng/mL and the intra- and interassay coefficients of variation for cortisol were 9% and 12%, respectively.

#### 2.2.2. Determining the Concentrations of 5-HT and Its Metabolite 5-Hydroxyl-Indole-3-Acetic Acid (5-HIAA) in the Pineal Gland

Pineal glands were analysed for 5-HT and 5-HIAA contents using high-performance liquid chromatography (HPLC) with electrochemical detection. Final concentrations were calculated relative to the recovery of the internal standard 5-hydroxyindole (Sigma-Aldrich, St. Louis, MO, USA) and expressed as pg/mg of tissue. The analytical procedures previously described by Smulikowska et al. [34] were used with minor modifications. Briefly, pineal glands were thawed and homogenised at 0°C in 0.1 M HClO_4_ (1 : 10  w/v) (Sigma-Aldrich, St. Louis, MO, USA) with 200 ng of 5-hydroxyindole added as an internal standard, and the homogenates were centrifuged in a SIGMA 1-14K centrifuge (Sigma-Aldrich, St. Louis, MO, USA) at 12 000 ×g for 15 min. The concentrations of 5-HT and 5-HIAA were determined via HPLC using a Waters 515 system (Waters Corporation, Milford, MA, USA) coupled to an electrochemical detector (Hewlett Packard 1049A, HP Inc., Palo Alto, CA, USA) equipped with a glassy carbon working electrode and a Ag/AgCl reference electrode. The electrochemical detector was set at an oxidative potential of 0.650 V. Supernatant (50 *μ*L) was injected into an LC-18-DB (15 cm × 4.6 mm ID, 5 *μ*m) Supelco column (Sigma-Aldrich, St. Louis, MO, USA) protected by a SUPELCOSIL LC-18-DB Supelguard 2 cm precolumn (5 *μ*m particle size; Sigma-Aldrich, St. Louis, MO, USA) and was eluded isocratically with a mobile phase consisting of 0.01 mol/L NaCl (Sigma-Aldrich, St. Louis, MO, USA), 0.001 mol/L EDTA (Sigma-Aldrich, St. Louis, MO, USA), and 12% CH_3_OH (Sigma-Aldrich, St. Louis, MO, USA). The pH of mobile phase was 3.6. The flow rate was set at 0.8 mL/min. The limit of detection was 10 pg/50 *μ*L for 5-HT and 5 pg/50 *μ*L for 5-HIAA.

#### 2.2.3. Isolation of mRNA and Protein from Pineal Glands

The total RNA and protein from the explants were isolated using the NucleoSpin® RNA/Protein Kit (MACHEREY-NAGEL Gmbh & Co., Düren, Germany). All steps of the isolation were performed according to manufacturer's protocol. The purity and concentration of the isolated RNA were spectrophotometrically quantified by measuring the optical density at 230, 260, and 280 nm in a NanoDrop 1000 spectrophotometer (Thermo Fisher Scientific Inc., Waltham, MA, USA). The RNA integrity was confirmed by electrophoresis using 1% agarose gel (Reducta NU, PRONA Marine Research Institute, Vigo, Spain) stained with ethidium bromide (Sigma-Aldrich, St. Louis, MO, USA).

#### 2.2.4. Real-Time PCR Assay

To synthesise cDNA, the Maxima*™* First Strand cDNA Synthesis Kit for RT-qPCR (Thermo Fisher Scientific, Waltham, MA, USA) and 2 *μ*g of total RNA were used. Real-Time RT-PCR was performed using the HOT FIREPol EvaGreen® qPCR Mix Plus (Solis BioDyne, Tartu, Estonia) and HPLC-grade oligonucleotide primers (Genomed, Warszawa, Poland). The primer sequences were designed using Primer 3 software [35, 36] (Table 1). One reaction mixture (total volume: 20 *μ*L) contained 4 *μ*L of PCR Master Mix (5x), 14 *μ*L of RNase-free water, 1 *μ*L of primers (0.5 *μ*L each primer, working concentration 0.25 *μ*M), and 1 *μ*L of the cDNA template. The reactions were run on a Rotor-Gene 6000 instrument (Qiagen, Dusseldorf, Germany). The following protocol was used: 95°C for 15 min and 30 cycles of 95°C for 10 s for denaturation, 60°C for 20 s for annealing, and 72°C for 10 s for extension. A final melting curve analysis was performed to confirm the specificity of the amplification.

Relative gene expression was calculated using the comparative quantification option [37] of the Rotor-Gene 6000 software version 1.7 (Qiagen, Dusseldorf, Germany). Three housekeeping genes were examined: glyceraldehyde-3-phosphate dehydrogenase (GAPDH), *β*-actin (ACTB), and histone deacetylase 1 (HDAC1). The mean expression of these three housekeeping genes was used to normalise the expression of the analysed genes. The results are presented in arbitrary units, as the ratio of the target gene expression to the mean expression of the housekeeping genes.

#### 2.2.5. Western-Blot Assay

Before electrophoresis, the protein concentrations of the samples isolated using the NucleoSpin® RNA/Protein Kit (MACHEREY-NAGEL Gmbh & Co., Düren, Germany) were quantified using the Protein Quantification Assay Kit (MACHEREY-NAGEL Gmbh & Co.; Düren, Germany). The appropriate volume of molecular grade water (Sigma-Aldrich, St. Louis, MO, USA) was added to a volume of sample containing 50 *μ*g of total protein to bring the total sample volume to 20 *μ*L. Next, to 20 *μ*L of these samples, 19 *μ*L of Laemmli buffer (Sigma-Aldrich, St. Louis, MO, USA) and 1 *μ*L of *β*-mercaptoethanol (Sigma-Aldrich, St. Louis, MO, USA) were added. These mixtures were boiled for 3 min. Electrophoresis was performed in the presence of molecular weight markers (Spectra Multicolor Broad Range Protein Ladder, Thermo Fisher Scientific Inc., Rockford, IL, USA). Denatured samples and molecular weight standards were loaded on 4–12% polyacrylamide gels and subjected to electrophoresis in a Tris-glycine running buffer using the Protean II xi Cell (Bio-Rad Laboratories, Inc., Hercules, CA, USA), according to the manufacturer's instructions. Next, proteins were transferred in Tris-glycine blotting buffer to polyvinylidene difluoride membranes (Immobilon*™*-P (0.45 *μ*m), Merck KGaA, Darmstadt, Germany) using the Trans-Blot® SD Semi-Dry Transfer Cell (Bio-Rad Laboratories, Inc., Hercules, CA, USA) for 30 min at 20 V. The membranes were blocked for 1 h at room temperature in blocking buffer made up of Tris buffered saline at pH 7.5 with 0.05% Tween-20 (TBST) (Sigma-Aldrich, St. Louis, MO, USA) containing 3% bovine serum albumin fraction V (Sigma-Aldrich, St. Louis, MO, USA). Next, the membranes were incubated overnight at 4°C with the following primary antibodies: goat anti-TPH polyclonal antibody (cat number sc-15116, Santa Cruz Biotechnology Inc., Dallas, TX, USA), rabbit anti-DDC polyclonal antibody (cat number sc-99203, Santa Cruz Biotechnology Inc., Dallas, TX, USA), goat anti-AANAT polyclonal antibody (cat number sc-55612, Santa Cruz Biotechnology Inc., Dallas, TX, USA), rabbit anti-HIOMT polyclonal antibody (cat number bs-6961R, Bioss Inc., Woburn, MA, USA), and mouse anti-ACTB monoclonal antibody (cat number sc-47778, Santa Cruz Biotechnology Inc., Dallas, TX, USA) dissolved in blocking buffer at dilutions of 1 : 100, 1 : 200, 1 : 100, 1 : 200, and 1 : 1000, respectively. After washing three times, the membranes were incubated with the following secondary HRP conjugated antibodies: bovine anti-rabbit IgG-HRP (cat number sc-2379, Santa Cruz Biotechnology Inc., Dallas, TX, USA), donkey anti-goat IgG-HRP (cat number sc-2304, Santa Cruz Biotechnology Inc., Dallas, TX, USA), and goat anti-mouse IgG1 heavy chain (HRP) (cat number sc-2304, Abcam, Cambridge, UK) dissolved in blocking buffer at a dilution of 1 : 10000. After washing three times, the membranes were visualised using chromogenic detection with a Pierce 1-step TMB-blotting substrate solution (Thermo Fisher Scientific, Waltham, MA, USA). After visualisation, the membranes were dried and scanned using an Epson Perfection V370 Photo scanner (Seiko Epson Corporation, Suwa, Japan). The densitometric analysis of the scanned membrane was performed using ImageJ software (Research Services Branch, National Institute of Mental Health, Bethesda, MD, USA).

### 2.3. Statistical Analysis

The statistical analysis was performed using the STATISTICA 10 software (StatSoft Inc., Tulsa, OK, USA). The raw data, after passing a normality test, were subjected to a two-way analysis of variance (ANOVA) to determine any significant influences of the two parameters (photoperiod and IL-1*β*-treatment), followed by Tukey's* post hoc* test. The results are presented as the mean ± SEM. Statistical significance was set at *P* < 0.05.

It should be noted that, for hormone and body temperature data, to identify treatment effects, the mean values for the period after the treatment (1 to 4 h) were established and included in the statistical analysis.

## 3. Results

### 3.1. Effect of IL-1*β* Icv. Injection on Rectal Body Temperature

**Figure 1:**
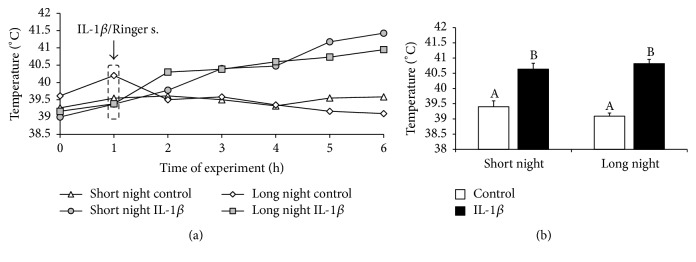
The effects of intracerebroventricular injection of interleukin- (IL-) 1*β* (50 *μ*g/animal) or Ringer's solution (50 *μ*L) on the rectal body temperature of ewes measured during the short night (SN, May/June) and long night (LN, November/December) photoperiods. Experiments started 1 hour after sunset. (a) presents the temporal patterns of rectal mean body temperature (*n* = 6 animals per group) measured in one-hour intervals. (b) depicts the mean (± SEM; *n* = 6 animals per group) rectal body temperatures for the period after IL-1*β* or Ringer's solution treatment (1 to 4 h) of ewes during the SN and LN photoperiods. Different capital letters indicate significant (*P* < 0.05) differences based on a two-way ANOVA followed by Tukey's* post hoc* test.

Central injection of IL-1*β* increased (*P* < 0.05) the rectal body temperature of ewes in the SN (40.6 ± 0.1°C) and LN (40.8  ±  0.2°C) photoperiods compared with that of the control groups (39.4  ±  0.2°C and 39.1  ±  0.1°C, resp.) (Figure 1).

### 3.2. Influence of IL-1*β* Icv. Injection on Melatonin and Cortisol Release

**Figure 2:**
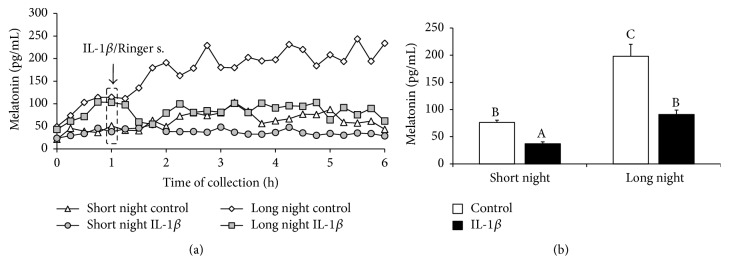
The effects of intracerebroventricular injection of interleukin- (IL-) 1*β* (50 *μ*g/animal) or Ringer's solution (50 *μ*L) on melatonin concentration in ewes taken during the short night (SN, May/June) and long night (LN, November/December) photoperiods. Experiments started one hour after sunset. (a) presents the temporal patterns of the mean concentration of circulating melatonin (*n* = 6 animals per group) in 15 min samples. (b) depicts the mean (± SEM; *n* = 6 animals per group) concentrations of melatonin for the period after IL-1*β* or Ringer's solution treatment (1 to 4 h) of ewes during the SN and LN photoperiods. Different capital letters indicate significant (*P* < 0.05) differences based on a two-way ANOVA followed by Tukey's* post hoc* test.

Control ewes from the SN photoperiod were characterised by lower (*P* < 0.05) levels of circulating melatonin (76 ± 4 pg/mL) compared with the levels in animals from the LN (198 ± 22 pg/mL) photoperiod. Central administration of IL-1*β* reduced (*P* < 0.05) secretion of melatonin in animals from both the SN (37 ± 3 pg/mL) and LN (91 ± 8 pg/mL) photoperiods (Figure 2).

**Figure 3:**
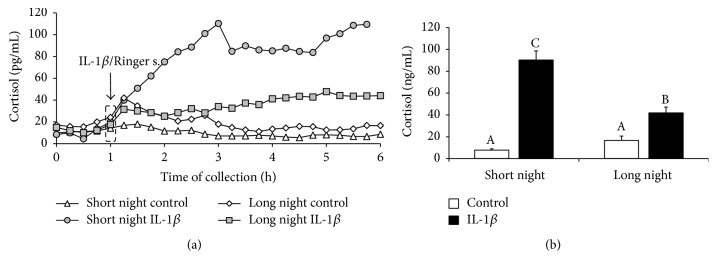
The effects of intracerebroventricular injection of interleukin- (IL-) 1*β* (50 *μ*g/animal) or Ringer's solution (50 *μ*L) on cortisol concentration in ewes taken during the short night (SN, May/June) and long night (LN, November/December) photoperiods. Experiments started one hour after sunset. (a) presents the temporal patterns of the mean concentration of the circulating cortisol (*n* = 6 animals per group) in 15 min samples. (b) depicts the mean (± SEM; *n* = 6 animals per group) concentration of cortisol for the period after IL-1*β* or Ringer's solution treatment (1 to 4 h) of ewes during the SN and LN photoperiods. Different capital letters indicate significant (*P* < 0.05) differences according to a two-way ANOVA followed by Tukey's* post hoc* test.

Treatment with IL-1*β* stimulated (*P* < 0.05) cortisol release in ewes from both the SN (90 ± 8 ng/mL) and LN (42 ± 5 ng/mL) photoperiods compared with its level in the control ewes (8 ± 1 ng/mL and 17 ± 4 ng/mL, resp.) (Figure 3).

### 3.3. Effect of IL-1*β* Icv. Injection on the Concentration of 5-HT and 5-HIAA in the Pineal Gland

**Figure 4:**
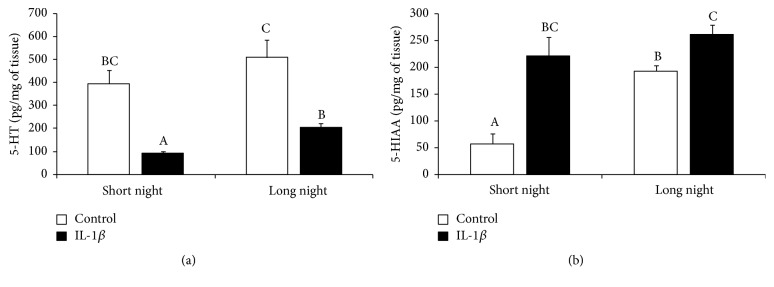
The effects of intracerebroventricular injection of interleukin- (IL-) 1*β* (50 *μ*g/animal) or Ringer's solution (50 *μ*L) on the mean (± SEM; *n* = 6 animals per group) concentration of serotonin (5-HT; (a)) and its metabolite 5-hydroxyl-indole-3-acetic acid (5-HIAA; (b)) in the pineal gland collected from control and IL-1*β*-treated ewes during the short night (SN, May/June) and long night (LN, November/December) photoperiods. Different capital letters indicate significant (*P* < 0.05) differences according to a two-way ANOVA followed by Tukey's* post hoc* test.

Injection of IL-1*β* into the third ventricle of the brain reduced (*P* < 0.05) 5-HT concentrations in the pineal tissue of animals from both the SN (91 ± 10 pg/mg) and LN (205 ± 17 pg/mg) photoperiods compared with its level in the control ewes (396 ± 59 pg/mg and 509  ±  77 pg/mg, resp.). Although the concentration of 5-HT in the tissues collected from the control animals did not differ, the reduction in 5-HT content was greater (*P* < 0.05) in the pineal glands dissected from the SN ewes than it was in the LN ewes (Figure 4).

It was found that the concentration of 5-HIAA in the pineal glands of control individuals was lower (*P* < 0.05) during the SN (57 ± 19 pg/mg) photoperiod than during the LN (193 ± 10 pg/mg) photoperiod. Central administration of IL-1*β* elevated (*P* < 0.05) the concentration of 5-HIAA in the pineal glands of ewes during both the SN (222 ± 35 pg/mg) and LN (261 ± 18 pg/mg) photoperiods (Figure 4).

### 3.4. Influence of IL-1*β* on the Relative Gene and Protein Expression of Enzymes of the Melatonin Biosynthetic Pathway

**Figure 5:**
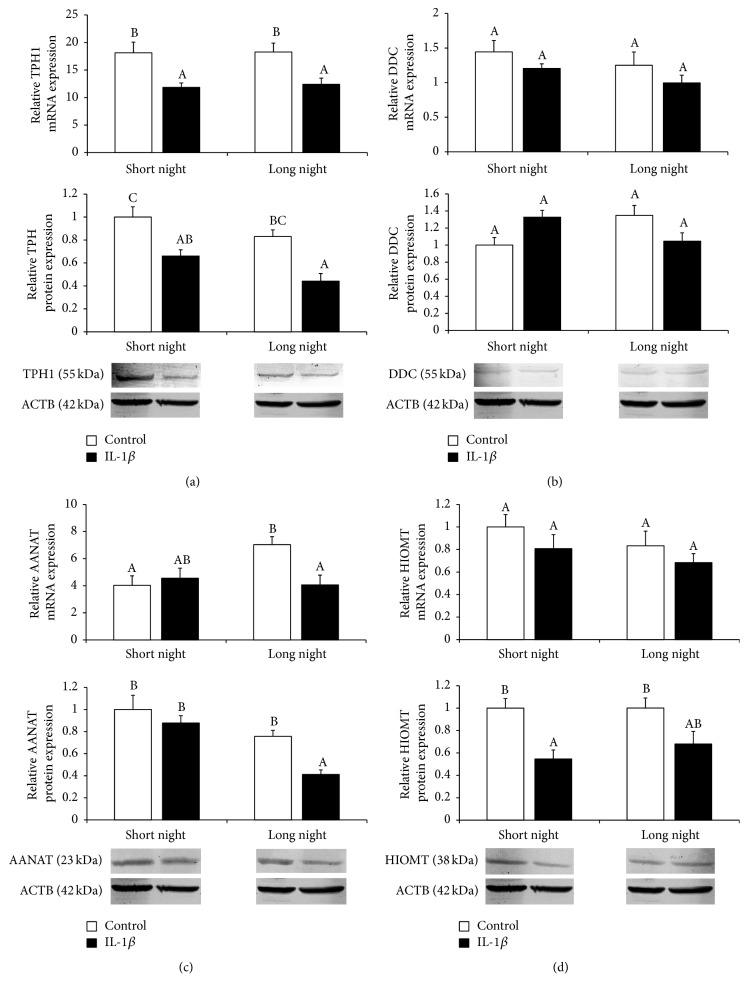
The effects of icv. injection of interleukin- (IL-) 1*β* (50 *μ*g/animal) or Ringer's solution (50 *μ*L) on the relative gene and protein expression (mean ± SEM; *n* = 6 animals per group) of enzymes of the melatonin biosynthetic pathway (tryptophan hydroxylase 1 (TPH1, (a)); aromatic-L-amino-acid decarboxylase encoded by the dopa decarboxylase (DDC, (b)); arylalkylamine-N-acetyltransferase (AANAT, (c)); hydroxyindole-O-methyltransferase (HIOMT, (d))) in ewes during the short night (SN, May/June) and long night (LN, November/December) photoperiods. All gene expression data presented in the figure were normalised to the average relative level of HIOMT gene expression in the control ewes from the SN photoperiod, which was set to 1.0. Protein expression data presented in a particular panel were normalised to the average relative level of protein expression in the control ewes from the SN photoperiod, which was set to 1.0. Different capital letters indicate significant (*P* < 0.05) differences according to a two-way ANOVA followed by Tukey's* post hoc* test.

Intracerebroventricular injections of IL-1*β* decreased (*P* < 0.05) the gene and protein expression of TPH enzyme in the pineal tissues collected from both SN and LN ewes (Figure 5(a)). IL-1*β* treatment suppressed (*P* < 0.05) the pineal expression of AANAT mRNA and protein but only in the glands of ewes from the LN photoperiod (Figure 5(c)). Moreover, icv. injection of IL-1*β* inhibited (*P* < 0.05) the expression of HIOMT protein but only in the pineal glands of ewes during the SN photoperiod. On the other hand, no effect of IL-1*β* treatment on the expression of HIOMT mRNA was found (Figure 5(d)). No influence of IL-1*β* icv. injection on the gene and protein expression of DDC was found (Figure 5(b)).

### 3.5. Effect of IL-1*β* on the Gene Expression of Proinflammatory Cytokines and Their Receptors in the Pineal Gland

**Figure 6:**
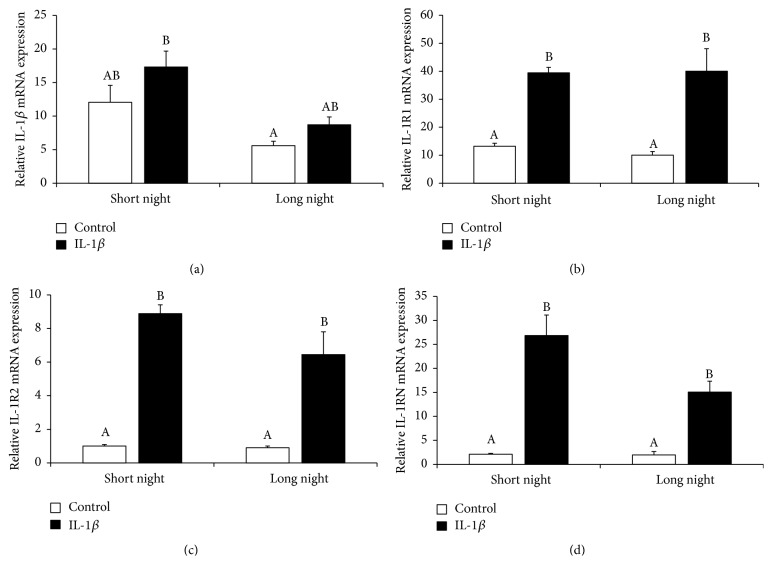
The effects of icv. injection of interleukin- (IL-) 1*β* (50 *μ*g/animal) or Ringer's solution (50 *μ*L) on the relative gene expression (mean ± SEM; *n* = 6 animals per group) of IL-1*β* (a), interleukin 1 type 1 receptor (IL-1R1, (b)), interleukin 1 type 2 receptor (IL-1R2, (c)), and interleukin 1 receptor antagonist (IL-1RN, (d)) in ewes during the short night (SN, May/June) and long night (LN, November/December) photoperiods. All data presented in the figure were normalised to the average relative level of IL-1R2 gene expression measured in the control ewes from the SN photoperiod, which was set to 1.0. Different capital letters indicate significant (*P* < 0.05) differences according to a two-way ANOVA followed by Tukey's* post hoc* test.

Injection of exogenous IL-1*β* into the third ventricle did not affect the gene expression of IL-1*β* in the pineal gland, but it stimulated (*P* < 0.05) mRNA expression of the IL-1 type 1 receptor (IL-1R1), the IL-1 type 2 receptor (IL-1R2), and the interleukin-1 receptor antagonist (IL-1RN) regardless of the photoperiods (Figure 6).

**Figure 7:**
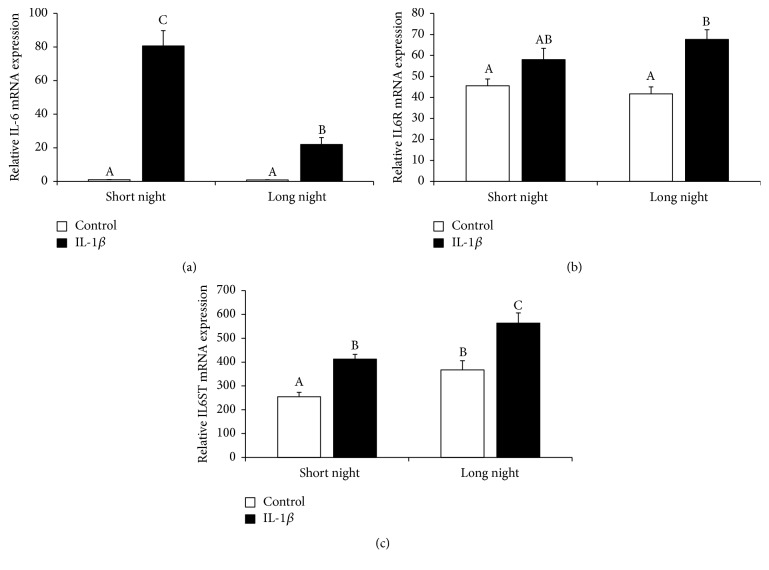
The effects of icv. injection of interleukin- (IL-) 1*β* (50 *μ*g/animal) or Ringer's solution (50 *μ*L) on the relative gene expression (mean ± SEM; *n* = 6 animals per group) of IL-6 (a), interleukin 6 receptor (IL6R, (b)), and interleukin 6 signal transducer (IL6ST, (c)) in ewes during the short night (SN, May/June) and long night (LN, November/December) photoperiods. All data presented in the figure were normalised to the average relative level of IL-6 gene expression in the control ewes from the SN photoperiod, which was set to 1.0. Different capital letters indicate significant (*P* < 0.05) differences according to a two-way ANOVA followed by Tukey's* post hoc* test.

IL-1*β* injection increased (*P* < 0.05) IL-6 gene expression. However, the action of IL-1*β* was stronger (*P* < 0.05) in the SN than in the LN photoperiods. In the animals receiving icv. injections of IL-1*β*, a stimulation (*P* < 0.05) of interleukin 6 signal transducer (IL6ST) gene expression was found regardless the photoperiod, and increasing (*P* < 0.05) levels of IL6 receptor (IL6R) gene expression were found but only in animals from the LN season (Figure 7).

**Figure 8:**
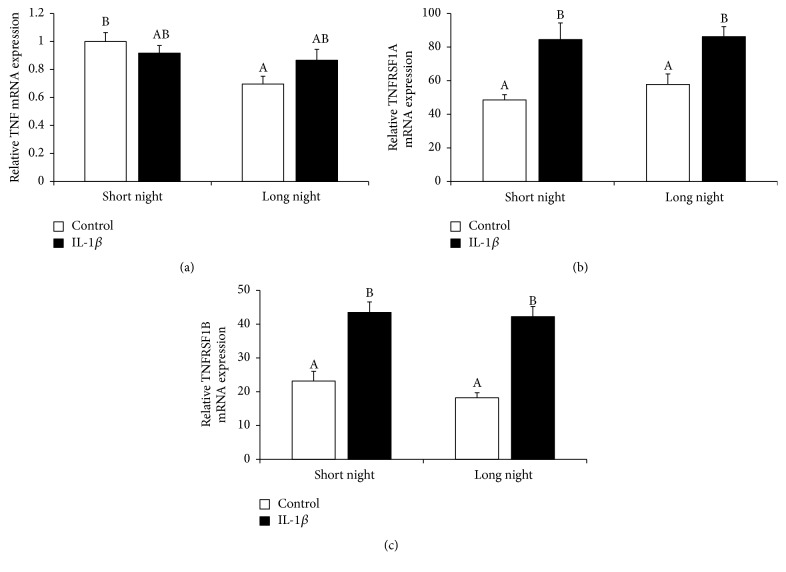
The effects of icv. injection of interleukin- (IL-) 1*β* (50 *μ*g/animal) or Ringer's solution (50 *μ*L) on the relative gene expression (mean ± SEM; *n* = 6 animals per group) of tumor necrosis factor *α* (TNF*α*, (a)), TNF type 1 receptor (TNFRSF1A, (b)), and TNF type 2 receptor (TNFRSF1B, (c)) in ewes during the short night (SN, May/June) and long night (LN, November/December) photoperiods. All data presented in the figure were normalised to the average relative level of TNF gene expression in the control ewes from the SN photoperiod, which was set to 1.0. Different capital letters indicate significant (*P* < 0.05) differences according to a two-way ANOVA followed by Tukey's* post hoc* test.

In was found that the nocturnal expression of mRNA encoding TNF was higher (*P* < 0.05) in the pineal glands from the SN animals than in those from the LN animals. Although IL-1*β* treatment did not influence the gene expression of TNF, its stimulatory (*P* < 0.05) effect on the expression of TNF type 1 receptor (TNFRSF1A) and TNF type 2 receptor (TNFRSF1B) mRNA was found, regardless of the photoperiod (Figure 8).

## 4. Discussion

The results presented in the paper represent the first study describing the effect of centrally acting IL-1*β* on the nocturnal secretion of melatonin from the pineal gland. It was clearly demonstrated that the injection of IL-1*β* into the IIIv suppressed melatonin release in sheep, regardless of the photoperiod. The data obtained here fully support the results of our* ex vivo* studies demonstrating that IL-1*β* suppressed NE-stimulated melatonin secretion from explants of ovine pineal glands [15]. The existence of an interplay between Il-1*β* and the pineal gland has also been found in experiments on rats indicating a significant dose-dependent decrease in serum melatonin levels under the influence of exogenous recombinant human IL-1*β*, which was abolished by an anti-human IL-1 receptor antibody [12]. Our study suggests that decreased secretion of melatonin may result from the reduced synthesis of its intermediate, 5-HT, in the pineal gland of IL-1*β*-treated animals. Moreover, the reduction in 5-HT concentration, which was higher during the SN than the LN photoperiod, fully reflects the changes in melatonin secretion. Lowering the pineal 5-HT concentration is not likely due to a reduction in tryptophan content in this gland. Numerous studies have shown that IL-1*β* noticeably increases the concentration of this amino acid in the brain [38–40]. The results obtained here suggest that the 5-HT decrease in the pineal gland may result, at least partially, from a decreased expression of the TPH1 enzyme in this gland. A reduction of the expression of the enzyme converting tryptophan to 5-HTP may profoundly influence all downstream stages of melatonin biosynthesis, especially because it was determined that TPH1 had the highest relative level of gene expression among the enzymes of the melatonin biosynthetic pathway. On the other hand, a reduction of pineal 5-HT levels may result from the increased metabolism of this neurotransmitter. The experiments performed on rodents also show that IL-1 treatment increases 5-HT metabolism throughout the brain [38, 39]. Our results show that central IL-1*β* injection decreased the concentration of 5-HT in parallel with increased concentrations of its metabolite, 5-HIAA. It is thought that 5-HT can be metabolised in the pineal gland via two distinct pathways. The first leads to the formation of N-acetylserotonin and then to melatonin. In the second one, 5-HT is metabolised by the enzyme monoamine oxidase (MAO) to yield 5-hydroxyindole acetaldehyde. In turn, this intermediate is then either oxidised to 5-HIAA or reduced to 5-hydroxytryptophol. Both of these are substrates for HIOMT and yield 5-methoxyindole acetic acid and 5-methoxytryptophol, respectively [41, 42]. Because norepinephrine (NE) was found as a factor inhibiting the formation of 5-HIAA in the pineal gland [41], increased concentrations of this metabolite suggest a reduced stimulation of the pineal gland by NE in IL-1*β* treated ewes. A study on rats showed that IL-1*β* stimulates the formation of the main NE metabolite 3-methoxy-4-hydroxyphenylethylene glycol in the central nervous system, which suggests that IL-1*β* promotes the metabolism of this neurotransmitter [39]. It is worth mentioning that the same study reported a decrease in the NE content of the hypothalamus, as well as in the dorsal posterior brain stem, after IL-1*β* treatment [39]. The expression of DDC seems to be constitutive and not sensitive to IL-1*β* treatment. However, the results obtained show a significant reduction of AANAT gene and protein expression in the pineal glands of individuals centrally injected with the cytokine during the LN photoperiod. In sheep from the SN photoperiod, no influence of IL-1*β* treatment on both gene and protein expression of AANAT was found. These results are generally consistent with our previous results obtained during an* ex vivo* study, which showed that IL-1*β* might have reduced the level of AANAT protein [15]. The modulatory action of IL-1*β* on AANAT expression seems to be targeted on the posttranscriptional level of the expression of this enzyme because the same study showed a lack of any effect of IL-1*β* treatment on AANAT mRNA expression. It is thought that, in all vertebrates, AANAT is the key enzyme in melatonin synthesis, often called the melatonin rhythm enzyme. It is currently postulated that the mechanisms regulating melatonin synthesis converge at the control of AANAT enzyme activity [43]. In all mammals studied, NE stimulates melatonin synthesis via the activation of two subtypes of adrenergic receptors, although the intracellular mechanisms leading to the stimulation of melatonin synthesis in the pinealocytes may vary among species. Activation of *β*1-adrenergic receptors increases the intracellular concentration of cAMP, which is followed by the activation of the cAMP-dependent protein kinase A (PKA). Both elevated cAMP levels and PKA activation are indispensable for the stimulation of AANAT and melatonin synthesis in all mammalian species studied so far. In turn, the activation of *α*1-adrenergic receptors elevates the intracellular calcium ([Ca^2+^]i) level caused by the release of calcium ions from intracellular stores [44]. The NE-dependent activation of the *β*1-adrenergic/cAMP/PKA and *α*1-adrenergic/[Ca^2+^]i pathways is conserved in mammalian physiology, but the downstream mechanisms that link these signalling cascades with AANAT activation and melatonin production exhibit marked interspecies variations [1]. In rodents, the cAMP/PKA pathway controls transcriptional mechanisms regulating melatonin synthesis. It has been demonstrated that stimulation by NE results in an approximately 100-fold increase in cAMP levels in rat pinealocytes [45]. Moreover, the day/night rhythm of the changes in cAMP concentrations in the rat pineal gland parallel the pattern of changes in AANAT mRNA expression, which increases by approximately 150-fold during the night [43, 46]. In ungulates and primates, melatonin synthesis is controlled by mechanisms targeting the posttranslational regulation of AANAT. In these animals, pinealocytes constantly synthesise the AANAT protein from continually available AANAT mRNA. In the absence of noradrenergic stimulation, the AANAT protein is destroyed by proteasomal proteolysis. Under NE stimulation, elevated cAMP levels result in the phosphorylation of AANAT by PKA. This posttranslational modification leads to the interaction of phosphorylated AANAT with 14-3-3 proteins, protecting AANAT from degradation [1, 47]. It is worth noting that our study shows that among all the genes for the enzymes of the melatonin biosynthetic pathway only AANAT gene expression exhibited a moderate but significant difference between the SN and LN photoperiods. To the best of our knowledge, this is the first scientific report showing that the nocturnal expression of the AANAT gene in ovine pineal glands may be affected by photoperiod. However, a previous study on sheep showed the presence of day/night fluctuations in AANAT transcription in the pineal gland. It was found that the nocturnal level of AANAT mRNA was approximately 2 times higher than the daily level of this transcript [48]. Some recent evidence suggests that HIOMT could also be a target for immune-pineal interactions. A study on chickens showed that peripheral inflammation enhanced both HIOMT gene expression and its enzymatic activity [4]. In addition, our* ex vivo* study on ovine pineal explants showed that glands collected from LPS-treated animals were characterised by higher HIOMT expression than those isolated from control ewes [15]. Because stress mediators are generally considered to be stimulators of melatonin secretion, it has been suggested that the stimulatory influence of inflammation on HIOMT expression and activity may result from inflammatory-dependent activation of the hypothalamic-pituitary-adrenal (HPA) axis activity [4]. Based on our results it might be concluded that HIOMT is not sensitive to IL-1*β* action at the transcriptional level, but IL-1*β* may reduce the protein expression of this enzyme. However, this suppressive effect of IL-1*β* on HIOMT was evident only during the SN period. This suggests that the influence of an immune/inflammatory challenge on HIOMT expression may not be the same under all circumstances.

The inhibitory action of IL-1*β* on the synthesis and release of melatonin may result from its direct action on the pineal cells. Our study showed that central injection of IL-1*β* increased the gene expression of both types of IL-1 receptor. It is generally accepted that IL-1*β* acts on target cells to stimulate the expression of its corresponding receptor [49]. In addition, our previous* ex vivo* studies showed that the direct action of IL-1*β* enhanced IL-1R1 gene expression in ovine pineal explants [15]. Moreover, the data presented here show that central IL-1*β* treatment significantly stimulates the transcription of IL-1RN in the pineal gland. It seems likely that increases in the gene expression of both IL-1R2 and IL-1RN in the pineal cells may be a mechanism protecting the gland against overstimulation by IL-1*β*. However, IL-1R2 is structurally similar to IL-1R1, and it plays a role as a decoy receptor. Its cytoplasmic domain is significantly truncated relative to that of IL-1R1 and is devoid of a Toll-IL-1 receptor (TIR) region, which renders IL-1R2 incapable of transmembrane signalling. In turn, IL-1RN is a competitive inhibitor that prevents IL-1*α* and IL-1*β* from interacting with IL-1R1 [50]. On the other hand, an increase in the amount of mRNA encoding IL-1R1 indicates a stimulatory effect on the transduction pathway of this receptor.* In vitro* studies have shown that the upregulation of IL-1R1 mRNA expression or cell surface levels of IL-1R1 by IL-1 is not an effect of its direct action. This study showed that the activation of the IL-1R1 transduction pathway by IL-1 leads to the stimulation of endogenous PGE_2_ synthesis, which in turn enhances IL-1R1 expression in the cells [49]. It is worth noting that the relative gene expression of IL-1R1 was higher than the expression of IL-1R2 and IL-1RN in the pineal gland from both the control and LPS-treated individuals, which indicates the importance of the IL-1R1-dependent signalling pathway in the regulation of pineal function. In addition, although the expression of the IL-1*β* gene was found in the ovine pineal gland, no influence of experimental treatment or photoperiod on the transcription of this cytokine was found. These data are consistent with our previous* ex vivo* study, which showed a lack of an effect of IL-1*β* treatment on its gene expression in pineal explants [15]. These results support previous reports on the constitutive expression of IL-1*β* in the rat pineal gland, which has also been shown to correlate with diurnal melatonin rhythms, where IL-1*β* mRNA levels are higher during daylight than during darkness [23]. Unexpectedly, this study found that IL-1*β* expression was upregulated in pineal cultures after treatment with NE. Increased IL-1*β* expression via NE treatment* ex vivo* and the decline in IL-1*β* expression at night, when NE levels are elevated, were explained based on immunocytochemical data showing that astrocytes are the predominant cell type expressing this cytokine* in vivo*, whereas IL-1*β*-positive cells are predominantly microglia in pineal explants and dispersed cell cultures. They assumed that these two types of IL-1*β* expressing cells may differently affect the pinealocytes activity. Our previous study performed on* ex vivo* model suggested that in sheep NE rather regulates pineal IL-1*β* synthesis via its anti-inflammatory mechanism of action, which was previously reported for other mammalian cells [51, 52].

Beyond the direct action of IL-1*β* on the pineal cells, this cytokine may also affect the melatonin synthesis indirectly via induction of the synthesis of other proinflammatory mediators. Its ability to stimulate the synthesis of proinflammatory cytokines such as IL-6 and TNF*α* has been well demonstrated in numerous cells [53]. Obtained results showed that icv. injection of IL-1*β* stimulated IL-6 gene expression directly in the pineal gland but the IL-1*β* induced transcription of IL-6 was higher in SN than LN photoperiod. The stimulatory influence of IL-1*β* on the expression of IL-6 has been previously described in the adult rat pineal organ cultures [24]. Our study showed that both mRNA encoding IL-6R and IL6ST are expressed in the ovine pineal gland. Moreover, IL-1*β* treatment stimulated the gene expression of IL6ST regardless of the photoperiod, and it stimulated IL-6R but only during the LN photoperiod. The expression of IL6-R mRNA has been previously demonstrated in the pineal gland of rats [54]. However, the same study showed that the transcription of IL-6R is rather stable and not sensitive to peripheral LPS treatment. These findings all suggest that ovine pineal cells may be sensitive to IL-6 stimulation; however, this issue has not been studied yet. Although central IL-1*β* injection did not influence TNF*α* transcription in our study, it significantly raised the gene expression of TNFRSF1A and TNFRSF2 in the pineal gland cells. This suggests that the pineal gland may be a target for TNF*α* synthesised in brain regions outside the pineal gland. It also might be that the lack of IL-1*β*-induced changes in TNF*α* transcription was a result of the time that elapsed since the icv. injection of IL-1*β*. The pineal glands were collected four hours after IL-1*β* treatment, and a study on rats, which analysed the profile of proinflammatory cytokines in the CSF after peripheral LPS injection, showed that, after initial elevation, the level of TNF*α* showed the fastest reduction among all assayed cytokines [20]. However, TNF*α* is known for its ability to interfere with the processes of melatonin biosynthesis. A study on denervated pineal glands in rats demonstrated that TNF*α* inhibited the transcription of AANAT and the synthesis of the melatonin precursor N-acetylserotonin [55]. Moreover, a study on women with acute inflammation showed that suppression of the nocturnal melatonin surge was significantly correlated with an increase in circulating TNF*α*. At the beginning of inflammation when TNF-*α* levels were high, the nocturnal surge of melatonin was suppressed, and as soon as TNF-*α* levels returned to levels that were below detection, the nocturnal melatonin surge was restored [56, 57]. It is worth mentioning that the results of our study analysing the gene expression of proinflammatory cytokines reflected the whole pineal gland, and it is impossible to judge which type of pineal cells expresses the mRNA encoding proinflammatory cytokines and their corresponding receptors. It is postulated that, in addition to direct actions on pinealocytes, cytokines and other immune factors regulate pineal gland functioning indirectly affecting the pineal glial cells [6]. It is considered that the effect of cytokines such as IFN-*γ* and IL-1*β* on pinealocytes is generally mediated by microglia, when TNF*α* exerts its biological effects acting directly on pinealocytes because TNF receptor is expressed directly on pinealocytes [21, 49]. It is worth mentioning that indirect action of IL-1*β* on the melatonin secretion could be also mediated by prostaglandins. However, in the present study, the effect of central injection of IL-1*β* on the brain prostaglandins has not been studied, the raise in the rectal body temperature in all treated ewes indirectly indicates that IL-1*β* stimulated production of central PGs. It was established that PGs are involved in the regulation of melatonin secretion in the pineal gland [13, 14]. However, the role of distingué PGs in the modulation of melatonin secretion seems to be differentiated. PGE_1_ and PGE_2_ increased melatonin secretion in rat pineal explants, whereas PGF_2*n*_ decreased melatonin release [13].

It has previously been shown that IL-1*β* administered both peripherally and centrally activates the HPA axis in several different species [58–60]. In the present study, icv. injection of IL-1*β* increased cortisol secretion, which indicates that the stress axis was activated. A study performed on rats showed that the pineal gland expresses glucocorticoid receptors, which potentiate NE-induced melatonin production in cultured rat pineal glands [61]. Additionally, results of an* in vivo* study on rats showed that a component of the HPA axis, corticosterone, enhanced nocturnal pineal melatonin production [54]. However, there is some evidence that the role of HPA axis components in the modulation of melatonin secretion is more complex and elusive. A study on humans demonstrated that corticotropin-releasing hormone has an inhibitory effect on the pineal secretion of melatonin [62]. However, IL-1*β* had a greater stimulatory effect on cortisol release during the SN than LN photoperiod. The lower stimulation of the HPA axis by central IL-1*β* may result from protective action of melatonin, whose circulating level is higher during the LN than during the SN photoperiod. Numerous studies have shown that melatonin attenuates the adrenocortical response to stress and reduces the biosynthesis, release, and glucocorticoid-responsiveness of hypothalamic adrenocorticotropic hormone secretagogues [63, 64].

## 5. Conclusion

In summary, our study supports the thesis that centrally acting IL-1*β* is responsible for distortions in nocturnal melatonin secretion patterns. This may result from a reduction of both serotonin concentration and the expression of the melatonin rhythm enzyme AANAT. Central IL-1*β* induces diverse pathophysiological responses in the central nervous system. Therefore, it is difficult to judge which IL-1*β*- induced pathway plays a pivotal role in the mediating of its inhibitory action on melatonin synthesis. It may involve mechanisms induced by direct action of IL-1*β* on the pineal cells through pineal IL-1 receptors. However, acting both at the pineal level and in other brain structures IL-1*β* may also provoke synthesis of other proinflammatory cytokines and PGs, which also may influence melatonin secretion. The indirect mechanism of IL-1*β* action on the pineal secretion of melatonin may also involve components of activated HPA axis; however the role of particular stress mediators in the modulation of melatonin secretion is differentiated. Of great importance is the fact that our research was performed on ewes. Till now, the majority of the studies concerning broadly understood bilateral interactions between immune and pineal systems were performed on rodents. However, there are important differences in the intracellular mechanisms regulating melatonin secretion that distinguished rodents and other mammals including ungulates and primates. It seems that obtained results may be considered as more universal for day-active species including human than the results of studies on nocturnal animals, such as rats. Therefore, our study may shed new light on the aetiology of melatonin secretion disorders, which commonly accompany inflammatory diseases, Alzheimer's disease, and depression.

## Figures and Tables

**Table 1:** All genes analysed by Real-Time PCR are listed with their full names and abbreviations.

GenBank acc. number	Gene	Amplicon size (bp)	Forward/reverse	Sequence 5′ → 3′	Reference
NM_001034034	GAPDH glyceraldehyde-3-phosphate dehydrogenase	134	Forward	AGAAGGCTGGGGCTCACT	Herman et al. [65]
			Reverse	GGCATTGCTGACAATCTTGA	

U39357	ACTB beta actin	168	Forward	CTTCCTTCCTGGGCATGG	Herman et al. [65]
			Reverse	GGGCAGTGATCTCTTTCTGC	

BC108088.1	HDAC1 histone deacetylase 1	115	Forward	CTGGGGACCTACGGGATATT	Herman et al. [65]
			Reverse	GACATGACCGGCTTGAAAAT	

XM_004016118	TPH1 tryptophan hydroxylase 1	128	Forward	CGTCCTGTGGCTGGTTACTT	Originally designed
			Reverse	TGGCAGGTATCTGGTTCTGG	

NM_173907.2	DDC dopa decarboxylase	150	Forward	TTCTTGCTTCGTGGTGGCTA	Originally designed
			Reverse	CGGAACTCAGGGCAGATGAA	

NM_001009461	AANAT aralkylamine N-acetyltransferase	154	Forward	CGAGAGGCCTTCATCTCTGT	Herman et al. [15]
			Reverse	GTCTCTCCTCATCCCACAGG	

KC290950	HIOMT hydroxyindole O-methyltransferase	167	Forward	AGCTTCCATGAAGGGGATTT	Herman et al. [15]
			Reverse	AGGAGGCTCTCGATGACCAG	

X54796.1	IL1B interleukin 1 beta	137	Forward	CAGCCGTGCAGTCAGTAAAA	Herman et al. [65]
			Reverse	GAAGCTCATGCAGAACACCA	

NM_001206735.1	IL1R1 interleukin 1 receptor, type I	124	Forward	GGGAAGGGTCCACCTGTAAC	Herman et al. [65]
			Reverse	ACAATGCTTTCCCCAACGTA	

NM_001046210.1	IL1R2 interleukin 1 receptor, type II	161	Forward	CGCCAGGCATACTCAGAAA	Originally designed
			Reverse	GAGAACGTGGCAGCTTCTTT	

NM_001308595.1	IL1RN interleukin 1 receptor antagonist	145	Forward	AGGATCTGGGATGTCAACCA	Originally designed
			Reverse	CATGGATCCCCAGGAACATA	

NM_001009392.1	*IL6* interleukin 6	165	Forward	GTTCAATCAGGCGATTTGCT	Herman et al. [65]
			Reverse	CCTGCGATCTTTTCCTTCAG	

NM_001110785	*IL6R* interleukin 6 receptor	149	Forward	TCAGCGACTCCGGAAACTAT	Herman et al. [65]
			Reverse	CCGAGGACTCCACTCACAAT	

XM_004016974	*IL6ST* glycoprotein 130	139	Forward	GGCTTGCCTCCTGAAAAACC	Originally designed
			Reverse	ACTTCTCTGTTGCCCACTCAG	

NM_001024860	*TNF* tumor necrosis factor	153	Forward	CAAATAACAAGCCGGTAGCC	Herman et al. [65]
			Reverse	AGATGAGGTAAAGCCCGTCA	

NM_174674	*TNFRSF1A* tumor necrosis factor receptor, type 1	137	Forward	AGGTGCCGGGATGAAATGTT	Herman et al. [65]
			Reverse	CAGAGGCTGCAGTTCAGACA	

NM_001040490	*TNFRSF1B* tumor necrosis factor receptor, type 2	122	Forward	ACCTTCTTCCTCCTCCCAAA	Herman et al. [65]
			Reverse	AGAAGCAGACCCAATGCTGT	
